# The fundamental units, processes and patterns of evolution, and the Tree of Life conundrum

**DOI:** 10.1186/1745-6150-4-33

**Published:** 2009-09-29

**Authors:** Eugene V Koonin, Yuri I Wolf

**Affiliations:** 1 National Center for Biotechnology Information, National Library of Medicine, National Institutes of Health, Bethesda, MD 20894, USA

## Abstract

**Background:**

The elucidation of the dominant role of horizontal gene transfer (HGT) in the evolution of prokaryotes led to a severe crisis of the Tree of Life (TOL) concept and intense debates on this subject.

**Concept:**

Prompted by the crisis of the TOL, we attempt to define the primary units and the fundamental patterns and processes of evolution. We posit that replication of the genetic material is the singular fundamental biological process and that replication with an error rate below a certain threshold both enables and necessitates evolution by drift and selection. Starting from this proposition, we outline a general concept of evolution that consists of three major precepts.

1. The primary agency of evolution consists of Fundamental Units of Evolution (FUEs), that is, units of genetic material that possess a substantial degree of evolutionary independence. The FUEs include both bona fide selfish elements such as viruses, viroids, transposons, and plasmids, which encode some of the information required for their own replication, and regular genes that possess quasi-independence owing to their distinct selective value that provides for their transfer between ensembles of FUEs (genomes) and preferential replication along with the rest of the recipient genome.

2. The history of replication of a genetic element without recombination is isomorphously represented by a directed tree graph (an arborescence, in the graph theory language). Recombination within a FUE is common between very closely related sequences where homologous recombination is feasible but becomes negligible for longer evolutionary distances. In contrast, shuffling of FUEs occurs at all evolutionary distances. Thus, a tree is a natural representation of the evolution of an individual FUE on the macro scale, but not of an ensemble of FUEs such as a genome.

3. The history of life is properly represented by the "forest" of evolutionary trees for individual FUEs (Forest of Life, or FOL). Search for trends and patterns in the FOL is a productive direction of study that leads to the delineation of ensembles of FUEs that evolve coherently for a certain time span owing to a shared history of vertical inheritance or horizontal gene transfer; these ensembles are commonly known as genomes, taxa, or clades, depending on the level of analysis. A small set of genes (the universal genetic core of life) might show a (mostly) coherent evolutionary trend that transcends the entire history of cellular life forms. However, it might not be useful to denote this trend "the tree of life", or organismal, or species tree because neither organisms nor species are fundamental units of life.

**Conclusion:**

A logical analysis of the units and processes of biological evolution suggests that the natural fundamental unit of evolution is a FUE, that is, a genetic element with an independent evolutionary history. Evolution of a FUE on the macro scale is naturally represented by a tree. Only the full compendium of trees for individual FUEs (the FOL) is an adequate depiction of the evolution of life. Coherent evolution of FUEs over extended evolutionary intervals is a crucial aspect of the history of life but a "species" or "organismal" tree is not a fundamental concept.

**Reviewers:**

This articles was reviewed by Valerian Dolja, W. Ford Doolittle, Nicholas Galtier, and William Martin

## Background

In Chapter 4 of *On the Origin of Species *[1], Darwin famously wrote:

"The affinities of all the beings of the same class have sometimes been represented by a great tree. I believe this simile largely speaks the truth. The green and budding twigs may represent existing species; and those produced during each former year may represent the long succession of extinct species. .... The limbs divided into great branches, and these into lesser and lesser branches, were themselves once, when the tree was small, budding twigs; and this connexion of the former and present buds by ramifying branches may well represent the classification of all extinct and living species in groups subordinate to groups."

Although the single figure in the Origin does not, exactly, depict that putative "great tree", but rather Darwin's ideas of evolution of species within genera, the fact that the only illustration in the book shows tree speaks volumes of the importance of the tree as a metaphor of evolution.

In the 6^th ^edition of the *Origin *[2], Darwin went further and explicitly introduced the notion of the Tree of Life (TOL):

*"As buds give rise by growth to fresh buds, and these, if vigorous, branch out and overtop on all sides many a feebler branch, so by generation I believe it has been with the great ****Tree of Life***, *which fills with its dead and broken branches the crust of the earth, and covers the surface with its ever-branching and beautiful ramifications."*

Since then, tree thinking in biology became standard and dominant. Starting with the seminal work of Haeckel [3], Darwin's abstract tree was populated with concrete life forms, and for the next century, the TOL was continuously refined as improving methods of analysis were applied to growing data collections. The 1960s and 1970s were marked by the birth and early advances of molecular phylogenetics that, unlike the traditional phenomic phylogenetics, deals directly with the evolving substrate, the sequences of genes (or proteins) [4,5]. Molecular phylogenetics reached maturity by the 1980s when rRNA was introduced as the universal phylogenetic marker, a development that led to numerous insights including the momentous discovery of Archaea, the third domain of life, the existence of which was not even suspected without the rRNA phylogenies [6,7].

Ever since Darwin and throughout the heroic age of molecular evolution, any phylogenetic tree was automatically assumed to represent the evolutionary history of the respective organisms - in other words, each such tree was construed as a "species" or "organismal" tree. This situation started to change when comparison of multiple, complete genomes of prokaryotes (archaea and bacteria) became possible towards the end of the last century. Horizontal gene transfer (HGT) that was discovered in the pre-genomic era but was generally considered a rare and non-consequential process (with some notable exceptions [8]) turned out to be a dominant mode of prokaryotic evolution [9-11]. Traditionally, HGT is inferred from discrepancies between the topologies of the phylogenetic tree for a given gene and the adopted species tree such as the rRNA tree, or by massive comparison of individual gene trees. The results of these comparisons show that, throughout the history of life, most likely, all genes experienced HGT, albeit with different frequencies [12-14].

Thus, in general, evolutionary histories of all genes are unique. Moreover, "highways" of HGT have been discovered, for instance, between hyperthermophilic bacteria and archaea [15-18]. Such strong preferences in HGT have the potential to produce evolutionary associations between organisms caused by processes other than vertical phyletic descent [12]. Taken together with the extensive gene loss and emergence of new genes (primarily, via duplication with subsequent rapid divergence), these discoveries show that genes possess unique individual histories that cannot be adequately described by any single tree.

Hence a crisis of the entire TOL concept. If the TOL is supposed to be a reflection of the history of all (cellular) life forms on earth, then, the discovery of the diversity of individual gene histories makes the concept obsolete and should lead to "uprooting" of the TOL [9,11,19]. Apart from the increasingly unreasonable attempts to deny the wide spread and major impact of HGT for evolutionary biology, there seem to be three cogent ways to deal with the TOL crisis that I list more or less in the order of their increasingly radical character [20,21].

1. Make trees for a set of (nearly) universal genes, preferably, several that are mostly coherent, and brand a consensus tree of these genes the TOL.

2. Search for a statistical central trend in the Forest of Life (FOL), that is, the entire compendium of individual gene trees, and denote the resulting tree the TOL.

3. Give up the TOL concept and with it the very distinction between vertical and horizontal gene flows, and replace tree analysis with the analysis of various types of evolutionary networks.

Here we attempt to outline a fourth path by arguing that tree representation of evolution is not only reasonable but is the only natural one because it is inseparable from the mode of replication of the genetic material, the key biological process that is both necessary and sufficient for evolution to occur. By contrast, species (organismal) tree does not appear as a fundamental concept because the fundamental, Distinct Unit of Evolution (FUE) is any genetic element with a unique evolutionary history, rather than an organism or a species. We submit that the appropriate depiction of evolution of life is the Forest of Life (FOL), that is, the sum total of trees for all FUEs. There seems to be a considerable degree of order in the FOL owing to coherent evolution of various groups of genes that comprise genomes or even species (in the case of sexually reproducing forms). Discovery and investigation of trends in the FOL is important for understanding evolution but it might not be necessary or even useful to denote any such trend the Tree of Life.

## The concept: Forest of Life as the natural description of life's history

### Replication is both the condition and the direct cause of evolution

The persistence and evolution of all life forms on earth is ensured by replication of nucleic acid molecules (represented by double-stranded DNA in all extant cellular organisms but by single-stranded DNA or RNA in numerous viruses). From an information-theoretical point of view, replication is the process of copying a one-dimensional string of symbols that is made possible by precise rules of correspondence between these symbols (base complementarity rules). Beyond doubt, replication is the single central biological process that ensures the continuity of genetic information transmission. It is less commonly realized, however, that replication not only makes possible but necessitates evolution, or more precisely, that evolution by random drift and natural selection is a simple and necessary consequence of error-prone replication. The interpretation of evolution as a straightforward consequence of genetic material replication was apparently first articulated by the famous Russian geneticists Kol'tsov and Timofeev-Ressovsky in the late 1920ies, long before the discovery of the base complementarity and DNA structure [22]. However, their prescient idea remained unnoticed, and it seems that the first direct and dramatic proof of this principle was given in the famous experiments of Spiegelman and coworkers on Darwinian evolution in a test tube [23,24]. Spiegelman and coworkers put genomic RNA of bacteriophage Qβ into conditions that were perfect for replication (excess of active replicase and substrates), and then, applied various selective pressures, the simplest one being limitation of the time available for replication by serial transfer of aliquots of the reaction mix to new test tubes. The drastic result of this experiment was that after only 75 passages the ~4 kilobase phage genome evolved into a mini variant consisting of about 400 nucleotides and retaining only the terminal sequences required for the recognition of the replicase [25]. Variation of the selective pressure (for instance, limited substrate concentration or inclusion of inhibitors) led to different evolutionary outcomes [26]. Thus, this bare minimum experimental setup provides for extremely efficient evolution by natural selection including drastic changes to the evolving molecules without any requirements other than the opportunity to replicate. Spiegelman's experiments were devised so as to ensure the dominance of selection but there is no doubt that, under different experimental conditions, evolution via genetic drift would have been prominent.

The obvious condition of evolution in Spiegelman-type experiments is accumulation of replication errors (mutations in the progeny nucleotide sequences) that generate variability, the material for selection (or drift). No process of information transmission can be error-free, a central staple of information theory that follows from the laws of thermodynamics [27]. Of course, it is, in principle, imaginable that the error rate of replication, although finite, would be so low that the great majority of replication cycles would occur with a 100% fidelity. However, this does not happen to be the case. The error rate of replication without special repair mechanisms, as seen in the simplest viruses including Qβ that was used in Spiegelman's experiments, is in the range of 10^-3^-10^-4 ^substitution per nucleotide which provides for close to one error per replication of an average viral genome [28]. Polymerases are also prone to other types of errors, such as slippage, which facilitate evolution by deletion as seen in Spiegelman's experiments or by duplication [29].

**Figure 1:**
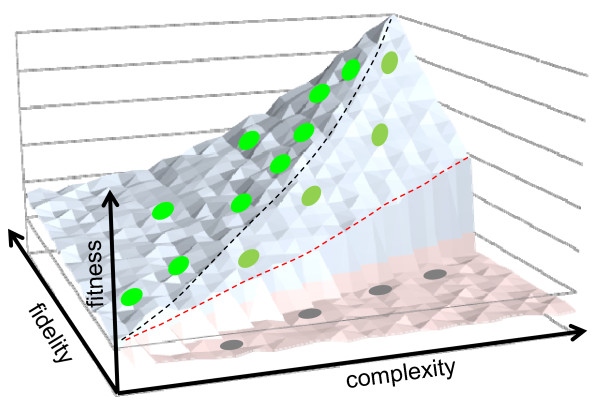
**Replicating genetic elements exist close to the replication error threshold**.

It is almost as intuitively clear that, although for evolution to occur, replication must be error-prone (and, replication is, in any case, error-prone owing to physical constraints), there must also exist an error threshold such that an above-threshold error rate renders evolution impossible. Extrapolating to the extreme (absurd), it is obvious that a "replication" process that incorporates nucleotides randomly is not conducive to evolution (and, of course, does not really qualify as replication). Spiegelman's experiments stimulated theoretical work by Eigen and coworkers that put the link between replication and evolution into a mathematical framework and quantified the requirements to the replication error rate [30]. Eigen's seminal work and subsequent, increasingly sophisticated analysis showed that the error threshold, that is, the minimal fidelity that is required for mutations to be fixed and, accordingly, for evolution to proceed, is relatively low, in the range of 1-10 errors per replication cycle (the exact number remains a matter of debate) [31-34]. It appears that most if not all replicating entities exist on or close to the edge of the "Eigen cliff", with the fidelity of replication only slightly exceeding the minimal requirement (Figure 1) [35].

An intriguing question is whether evolution involves "selection for evolvability" [36,37] or the existence near the edge results from the opportunistic character of the evolutionary process whereby fidelity is increased to the extent strictly necessary but not far beyond that because further increase would incur substantial cost of selection. However, discussion of this important problem is beyond the scope of this article.

The error threshold, certainly, is a crucial issue for the study of the origin of replication - and evolution. There is a distinct sense of paradox here because high replication fidelity hardly can be achieved without a complex replication machinery but such a machinery cannot evolve sufficiently accurate replication. Conceptually (but not necessarily in practice) the paradox is resolved by the "Darwin-Eigen cycle" in which a mutation leading to an increase in the complexity of the replicase is fixed if it leads to increase in fidelity that is greater than that required to replicate the evolved version of the replicase, and this increased fidelity in turn allows further growth of complexity [35,38]. Again, we only need to articulate this problem here but not discuss the details.

Summarizing the discussion of the intrinsic link between replication and evolution in what we would like, with deliberate arrogance, denote "the Central Dogma of evolution" (Figure 1):

-replication of a nucleic acid with the error rate below the error threshold necessarily leads to evolution by drift and natural selection

-replication with the error rate above the threshold leads to evolutionary dead end and eventually extinction of the respective genetic elements

-(replication with an error rate too low to allow evolution does not seem to be a practical issue).

### A tree as an isomorphous representation of replication history

**Figure 2:**
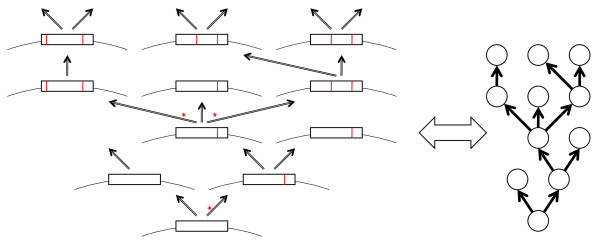
**A tree (arborescence) as an isomorphous representation of the error-prone replication process**. An idealized schematic of the replication history of a genetic element that includes both bifurcations and a multifurcation (shown by asterisks). Fixed mutations are shown by red strikes.

In the preceding section, we presented the concept of evolution being a direct consequence of error-prone (but not too erratic) replication. Here we emphasize that replication and the ensuing evolution are inherently tree-like processes: a replicating molecule gives rise to two (in the case of semi-conservative replication of dsDNA that occurs in all cellular organisms and many viruses) or multiple (in the case of the conservative replication of viruses with ssDNA or ssRNA genomes) copies with errors, resulting in a tree-like process of divergence (Figure 2a). In graph-theoretical terms, such a process can be isomorphously represented by a specific form of a directed acyclic graph known as arborescence that is a generalized tree in which multifurcations are allowed and all edges are directed away from the root [39](Figure 2b). A deviation from this simple pattern is occasional extinction of one or both progeny molecules; however, the resulting graph in which some of the vertices emit no edges does not violate the definition of an arborescence (Figure 2ab) (hereinafter, in order to stick to commonly used terms, we speak of trees rather than arborescences).

**Figure 3:**
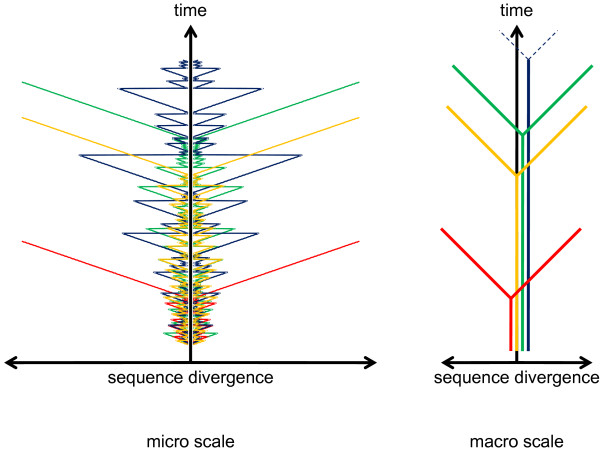
**Evolution of a FUE is reticulate on the micro scale but tree-like on the macro scale**. The cartoon schematically depicts the evolution of 4 FUEs shown by different colors. The divergence history of each FUE was simulated under the model of random homologous recombination, with the probability of recombination exponentially decreasing with sequence divergence. At each simulation step, the two daughter FUEs diverge by a constant amount (clock-like divergence) and either undergo homologous recombination (which brings the difference between the two back to zero) or not, preserving the existing state of divergence. After a number of short periods of divergence and recombination, the FUEs stochastically diverge far enough for recombination to become extremely unlikely after which point they continue diverging without recombination. At a macro scale this appears as a simple bifurcation in the tree-graph.

A major potential complication to the tree-like character of evolution is recombination that, if common, would turn the tree-like representation of the history of a replicating lineage (Figure 2b) into a network. Is it possible to determine a fundamental, "atomic" level of genetic organization at which recombination is negligible? This does not seem to be feasible in the case of homologous recombination that is extensive during coreplication of closely related sequences, in particular, in eukaryotes that engage in regular sex, and in "quasi-sexual" prokaryotes [40-43]. Essentially, the unit of homologous recombination is a single basepair. In contrast, homologous recombination cannot occur between distantly related sequences, so HGT between diverse prokaryotes involves only non-homologous (illegitimate) recombination complemented by more specific routes such as dissemination via bacteriophages and plasmids. In contrast to homologous recombination, a strong preference for evolutionary fixation of non-homologous recombination events outside genes or between parts of genes encoding distinct domains of multidomain proteins should be expected because preservation of gene integrity after non-homologous recombination is extremely unlikely. The prevalence of intergenic recombination in the course of HGT between distantly related prokaryotes has not been studied in sufficient detail. Nevertheless, a recent study shows that, at least, regions encoding relatively small domains are significantly avoided by recombination [44]. Thus, the evolutionary history of a gene or domain is reticulate on the micro scale owing to homologous recombination but is, largely, tree-like on the macro scale (Figure 3).

It has been argued and demonstrated on compelling examples that a tree can well describe relationships that have nothing to do with common descent, so "tree thinking" was deemed not to be *a priori *relevant in biology or, in the very least, not necessarily central in biology [19]. Although valid in itself, this argument seems to miss the crucial point discussed above, namely, that a tree is a necessary formal consequence of the descent history of replicating nucleic acids and the ensuing evolution. Thus, trees cannot be banished from evolutionary biology for a fundamental reason: they are intrinsic to the evolutionary process. This being the case, the main pertinent question becomes: what are the fundamental units whose evolution should be represented by trees? In the practice of evolutionary biology, trees are most often built for individual genes or for sets of genes that are believed to evolve coherently. However, it is typically implied (or even stated explicitly) that the ultimate goal is a species (organismal) tree. We believe that the lack of clarity about the basic unit to which tree analysis applies is the source of the entire TOL controversy.

### Distinct units of evolution: the fundamental agency of (tree-like) evolution

Conceptually, the answer to the question posed at the end of the preceding section seems clear: the fundamental unit of evolution can be most adequately defined as the smallest portion of genetic material with a distinct evolutionary trajectory, that is, evolving independently of other such units at least during some periods of evolution. We would like to introduce the notion of Distinct Units of Evolution (FUE) whose key characteristic is the potential of differential reproduction that makes them subject to selection independently of other FUEs.

It seems useful to differentiate two classes of FUEs:

i) bona fide selfish elements such as viruses, viroids, transposons, and plasmids - all these elements encode some of the information required for their replication and are united through their ability to promote their evolutionary success by exploiting resources of other organisms [45]

ii) quasi-independent elements that do not encode devices for their own replication but possess distinct selective value and, in that capacity, can be transferred between ensembles of FUEs (genomes) and promote their own replication along with the rest of the genome - essentially, any functional gene or even a portion of a gene encoding a distinct protein domain with an independent functional role fits this definition.

The concept of FUEs obviously borrows from the "selfish gene" idea of Dawkins [46] and the selfish operon hypothesis of Lawrence and Roth [47,48]. These concepts might generate a degree of confusion by assigning "selfishness" to genetic elements that do not actively contribute to their own replication at the mechanistic level. It seems that the partitioning of FUEs into two distinct classes eliminates this tension.

Of course, like any definition, the definition of a FUE meet its share of difficulties when one considers that many of the FUEs of the first kind consist of multiple FUEs of the second kind. Nevertheless, the distinction between the two types of FUEs seems an important one to make, given the major effect the selfishness of the first type of FUEs has on their evolutionary trajectories. Furthermore, the notion of a FUE can appear murky when one considers that even small functional elements such as promoters or enhancers, under some circumstances, have the potential of behaving like FUEs. Nevertheless, we believe that this concept is fundamental to our understanding of evolution. Indeed, given the extensive HGT that dominates the prokaryotic world, any gene or a portion of a gene encoding a distinct domain possesses a degree of independence and can be fixed in the recipient population even if the conferred advantage is relatively small, or even neutrally [49]. Therefore the prokaryotic genetic universe is most appropriately viewed as consortium of FUEs with varying degrees of independence some of which form ensembles that evolve over extended time intervals and are more commonly known as genomes of viruses, plasmids, and cellular life forms [50].

Perhaps, an even stronger motivation for the FUE concept comes from the theoretical research and simple logical considerations on pre-cellular evolution. It appears outright inconceivable that the first replicating elements were comparable in size and complexity to modern prokaryotic genomes. Instead, evolution of life must have started with ensembles of relatively small FUEs some of which would provide means for the replication of others that in turn would provide other benefits such as precursor synthesis, resulting in symbiotic relationships; other fully selfish elements would necessarily parasitize on such ensembles. Physical joining of FUEs would have been beneficial in many cases, provided sufficient replication fidelity, so the maximum size of a FUE would increase in the course of evolution. Qualitative and quantitative models of this collective phase of life's evolution have been developed [35,51-54]. In particular, we argued that this pre-cellular stage of evolution could be considered virus-like in many respects, and that the principal classes of extant viruses and other selfish elements emerged already at that stage [45,55]. There is an ongoing debate with regard to where in the history of life this collective stage belongs, and in particular, whether or not the Last Universal Cellular Ancestor (LUCA) was a typical cell, a cell with a fragmented genome, or a pre-cellular ensemble of genetic elements [52,56,57]. The discussion of the arguments pro and contra each of these models is beyond the scope of the present article but, in principle, there is no reasonable doubt as to the reality of the collective stage. Furthermore, it is often and reasonably argued that extensive mixing and matching of FUEs (that may or may not be called HGT depending on whether or not this stage is envisaged as cellular) was not only an inherent feature of this evolutionary stage but also a pre-requisite of a rapid increase in genetic and organizational complexity of life forms [45,52,55,58,59].

Considering the virtual inevitability of an early collective stage of evolution and the extensive HGT that permeates modern prokaryotic world, it stands to reason to view the entire evolution of prokaryotes as a dynamic process that plays out on the network of FUEs although relatively stable genomes consisting of hundreds and thousands of FUEs, of course, are major components of that network [50]. This view of the prokaryotic world implies that FUEs are fundamental units of evolution whereas all other levels of genetic organization are best considered to be derived.

The dominant pattern of evolution of eukaryotes, at least, of multicellular plants and animals is different: genomes are much more stable so that congruent evolution of large set of genes (FUEs) is the rule, and a species tree can be viewed as a (more or less) natural construct. However, evolution of unicellular eukaryotes, especially, those with a phagotrophic lifestyle, involves substantial levels of HGT [60,61], and even more strikingly, evolution of eukaryotes is a series of endosymbiotic events, from the primary mitochondrial endosymbiosis to a variety of secondary ones including the acquisition of the chloroplast by the common ancestor of plants and algae [62,63]. Moreover, mobile selfish elements make tremendous contributions to the evolution of eukaryotic genomes [64]. Thus, in our view, the only fundamental unit of evolution, both in prokaryotes and in eukaryotes, that can be defined without internal contradictions is a FUE although long stages of evolution of major groups of organisms certainly exist for which the concept of a species tree - and species as such - makes sense.

### The Forest of Life and trends in it

If (i) FUEs are fundamental units of evolution, whereas all other levels of organization of the genetic material including genomes are derived, and (ii) a tree is a necessary form of description of the evolution of any FUE, then, the adequate representation of evolution of life as a whole is the full compendium of FUE-specific trees that can be conveniently denoted the Forest of Life (FOL). This being the case, the notion of a species tree becomes, if not obsolete, at least secondary, applicable to some phases of evolution of some groups of organisms but not in general, hence not particularly important. This conclusion is not to be taken as an indication that there is no order in the FOL, and that signals of coherence among the trees are not to be sought. Such patterns are indeed discernible [65], and the central trend, even if relatively weak, seems to correspond to the signal of apparent vertical inheritance detectable in the phylogenies of genes encoding components of the translation machinery [66]. There seem to be other trends in the FOL as well, some of these, most likely reflecting preferential routes of HGT. A comprehensive exploration of the FOL is the primary goal of phylogenomics.

## Conclusion

A logical analysis of the units and processes of biological evolution suggests that:

-replication is the central, most fundamental process in biology;

-error-prone replication is both necessary and sufficient for evolution via drift and natural selection;

-the natural fundamental unit of evolution is a FUE, that is, a genetic element with an independent evolutionary history;

-the natural and necessary representation of evolution of a FUE on the macro scale is a tree (more precisely, an arborescence).

A corollary of these simple propositions is that the proper representation of the history of life is the entire Forest of Life. Search for order in the FOL can reveal important trends explained either by congruent vertical evolution of sets of FUEs of varying size, or by "highways" of HGT. In contrast, the quest for an all-encompassing TOL is futile. Darwin was entirely correct in his belief that a tree is an accurate depiction of the evolutionary process but, for obvious reasons, he was unable to correctly identify the fundamental unit of evolution.

## Authors' contributions

EVK initiated the project and wrote the initial draft of the paper; YIW designed and produced the figures; EVK and YIW jointly wrote the final version of the manuscript.

## Reviewers' comments

### Reviewer 1: Valerian Dolja, Oregon State University

This is another installation in the series of Koonin solo and Koonin & Wolf duo papers dealing with the most fundamental and hotly debated problems of evolutionary genomics. It is very timely, incisive, cool, pragmatic, certainly, not "the final truth", but about the best that can be offered at the moment. It also helps that the style of the paper shows enviable intellectual candor and clarity. Although I am not directly involved in the TOL business, I felt compelled to review this paper even from a prospective of a virologist interested in the big picture of life origin and evolution.

One conclusion of the essay, that the pursuit of a 'simple and true' organismal TOL is over due to overwhelming contributions of HGT to the life histories of viruses, prokaryotes, and unicellular eukaryotes, the organisms that together represent the substantial majority of genetic universe, is the only conclusion fully compatible with the results of comparative genomics. Another major conclusion, that evolution of individual genes is faithfully represented by trees, whereas evolution of organisms (or, preferably, genomes) can only be rendered as a FOL, also seems to be an inevitable outcome of the presented logical analysis of the units and processes of molecular evolution. Importantly, these conclusions provide a sensible and constructive alternative to the fierce and increasingly futile argument about the viability of the TOL.

Having said this, I would like to comment on two particular aspects of the analysis offered by Koonin and Wolf.

1. After going through two incarnations of the work, I am not yet at peace with the FUE definition. I would prefer defining FUE as a genomic unit encoding protein, protein domain, structural or regulatory RNA, or a control element with independent evolutionary history. [Evolutionary Unit of a Genome, or EUG]. I do not see a need in categorizing FUEs into selfish elements such as viruses on one hand, and regular genes on the other. First, selfish elements, with the exception of viroids, are ensembles of 'regular genes' even though some of them encode replication-related functions. Second, some of the regular genes of the 'non-selfish', that is, cellular genomes, are also involved in replication. Third, both selfish elements and cellular genomes are collections of genes of various 'selfishness quotient', all of which are equal subjects to HGT. Therefore, both cellular and noncellular genomes represent the same genetic continuum that can be analyzed in its entirety using the same rules and approaches.

Authors' response: *We do not really see any contradiction between the FUE definition and the EUG definition suggested by Dolja (we do appreciate the EUphony of the latter). As for partitioning of the FUEs into the selfish and non-selfish classes, we tend to believe that the validity and usefulness of this classification is an open question that is subject to quantitative analysis using an appropriate mathematical framework and the available extensive comparative-genomic data. Indeed, any FUE, in principle, can be assigned a 'selfishness quotient'. However, the correct formula for this quotient let alone the distribution of its values across FUEs remain unknowns. Once this formula is derived and the distribution is drawn, the issue of the classification of FUEs will become tractable. If the distribution turns out to be bimodal, the two classes will be rectified; if by contrast, the distribution is unimodal, the continuum model will be more appropriate. This problem is the target of an ongoing investigation in our group*.

2. I am not sure I can fully agree with the statement that the FUE rather than the organism or species (or, better, the genome) is the only fundamental agency of evolution. The genome is, indeed, a true unit of replication, and as such, also has a unique evolutionary history. Focusing solely on FUEs leaves behind the entire realm of population genetics that, as groundbreaking work of Michael Lynch has demonstrated, is one of the major determinants of the evolution of genome architecture. In other words, the laws of genome evolution are not reducible merely to sum total of FUEs' evolutionary histories because how the ensemble of FUEs put together depends in large part on population dynamics that is not determined by FUEs per se.

Authors' response: *We do not agree with such a categorical statement either and do not believe that it is contained or even implied in this article. Genomes are also fundamental and so are populations of organisms. However, in some ways, in particular, with respect to evolutionary independence and the applicability of trees as models of evolution FUEs appear...well, more equal than those other units. As for the groundbreaking work of Lynch, this is a bit ironic but a good idea to clarify. Lynch actually emphasizes that the effective population size, even in eukaryotes, where horizontal transfer is not much of an issue, the effective population size (the key parameter of the theory) is, generally, different for different genes. So we are not at all leaving behind population genetics which is indeed crucial for understanding evolution at any level - it seems that the true unit of evolution within the population-genetic framework is...a FUE*.

### Reviewer 2: W. Ford Doolittle, Dalhousie University

Koonin and Wolf aptly present one of the several possible responses to the Tree of Life kerfuffle, although their paper won't likely make the conflict just go away, as they and many readers no doubt wish. What these authors hope to do is displace the Tree of Life with a "Forest of Life" as the all-embracing metaphor for the history of living things. This is progress within the paradigm, to be sure, and I think they are right to point out, for instance, that because homologous recombination falls off as gene sequences diverge but lateral gene transfer uses mechanisms that are indifferent to gene sequence, gene evolution is inherently tree-like while genome evolution is not.

What I am less sure of is that we really need to "define the primary units and the fundamental patterns and processes of evolution". It is this kind of deep metatheoretical approach that gave us the Tree of Life in the first place and that keeps us on the hotseat in the ongoing struggle against the proponents of intelligent design and other nonsensical nonscience. Really, we understand pretty well all the individual genetic, population, ecological and environmental mechanisms that effect the evolution of genes, genomes, organisms and species, and we waste our times (and make ourselves vulnerable to creationist pettifoggery) by imagining there are causal processes or patterns deeper than these that we need to conceptualize. (The introduction of graph theoretical language really does not get us any closer to some fundamental truth, I think.)

It may well be sensible to talk about Fundamental Unit (s) of Evolution (FUE), but authors have a rather limited range of such units they will consider, and perhaps too much faith in their boundedness. Organisms, populations and even species can be FUEs, and over their own appropriate time scales are every bit as much "fundamental units of life" as are genes, while all sorts of entities in between can "evolve coherently for a certain time span." Again, I question the sense of describing one level or unit as more fundamental than another, but such is common practice and no reason not to consider this a valuable contribution in that tradition. So the rest of my remarks will be quibbles about specifics.

Authors' response: *We realize the dangers (and the seductions) of metanarratives *[67]. *And yet, we also tend to think that, in different domains of physical reality, there are distinct levels that are central to the adequate description and "understanding" of each domain. Molecules are fundamental to (bio)chemistry, cells to physiology (broadly construed), stars to astrophysics, galaxies to cosmology. In the same sense, we maintain that our FUEs are fundamental units of evolution which by no means applies that other levels are unimportant*.

1. In my copy of the Origin, its only figure appears eight pages *before *the quoted passage, and is clearly meant to illustrate evolution of the "species of a genus large in its own country", not the great tree 'simile" whose veracity Darwin accepted. The quote does not "introduce" the figure. Not that this should really matter: it's remarkable the extent to which accurate readings of Darwin seem essential to the credibility of current theory, as if evolutionary biology actually were the quasi-religious enterprise our opponents treat it as.

Authors' response: *although there is absolutely no religious bend in our attitude to Darwin (or anyone/thing else), we do maintain that the *Origin *is, possibly, THE greatest book ever published, so precision is highly desirable when it is discussed, and correction much appreciated. The respective text was modified to eliminate any misrepresentation*.

2. The Spiegelman experiments were certainly exciting and important (and I had the good fortune to have summer jobs in Sol's lab while they were going on). But I think that what impressed the community about these experiments was that they actually worked, and so well, rather than that they proved that evolution could occur as "straightforward consequence of genetic material replication". Probably no one doubted that.

Authors' response: *Seems to be a bit of a semantic issue. Perhaps, no one questioned evolution "in principle"...but few believed it would work "so well". We emphasize this aspect in the revision: "including drastic changes to the evolving molecules"*.

3. I don't think we (ref. [19]) really meant that tree-thinking is not relevant in biology, but rather that it was only one way to think about biology.

Authors' response: *we wrote "not a priori relevant" which makes a difference. In any case, in the revision, it is "so "tree thinking" was deemed not to be a priori relevant in biology or, in the very least, not necessarily central in biology"*.

4. The authors' view (which I share) that all or most all gene families have different evolutionary histories entails that the different last common ancestral versions of the different gene families existed at different times in the past, in different genomes. There was no LUCA, if by that we mean a single cell whose genome contained the ancestors of all modern genes. It really makes no sense to think of a population as "the ancestor", which is what I think authors mean by the statement "there is no reasonable doubt as to the reality of the collective stage". Today's cells comprise a population that derives from successively earlier populations, but there is no principled way to designate any one of these as LUCA.

Authors' response: *Actually, we largely agree with this position on LUCA *[45,52]* and argue elsewhere that it is probably more appropriate to speak of LUCAS, the Last Universal Common Ancestral State *[55,68]. *Here, however, we were disinclined to take a strong stance on this issue that is not really the subject of the article*.

### Reviewer 3: Nicolas Galtier, CNRS - Université Montpellier 2

This paper exposes the pros and cons of using trees to represent evolution, especially in prokaryotes. Genes or gene portions are defined as fundamental evolving entities (FUEs), and the relationship between gene trees, species trees, and the "tree of life" are discussed. I liked the direct and provocative tone of the text, and the clarity of the viewpoint newly expressed.

#### - Statements with which I fully agree

*1. Evolution results from replication with error*.

*2. Trees are a natural, useful representation of evolving molecules (genes)*.

(but see comment 5)

*3. The forest of gene trees is to be searched for patterns and trends*.

*4. The central trend has a strong, obvious meaning in animals and plants (if one appropriately accounts for endosymbiosis), less clearly so in microorganisms, depending on the prevalence of horizontal gene transfer*.

#### - Statements which I think could deserve additional empirical support

*5. Within-gene nonhomologous recombination (i.e., HGT of pieces of genes) is rare*.

I am not sure this issue has been examined with the necessary care. People typically build gene trees, without systematically checking potential conflicts between portions of genes.

Authors' response: *It is true that this has not been empirically studied with the necessary care and detail, and in the revised manuscript we changed the language to reflect this. We also cite a recent study that at least demonstrates preferential recombination outside regions encoding relatively small domains *[44].

6. There are substantial levels of horizontal gene transfer in unicellular eukaryotes

I'm not sure which studies this statement refers to, and which taxa are concerned.

Authors' response: *There is actually considerable amount of data on different unicellular eukaryotes. As it is not our intention here to get into the details on this particular subject, in the revised text, we cite two review articles that summarize the detected instances of HGT in unicellular eukaryotes *[60,61]

#### - Statements with which I am not sure I agree

7. Genes as the fundamental evolving unit

The definition of FUEs (Fundamental Units of Evolution) is the most original, and perhaps controversial, aspect of this manuscript. It is proposed that not only self-replicating genes, but also regular genes (or protein domains), should be considered the basic evolutionary entities, genomes (species) being seen as transient groups of genes of little evolutionary relevance. Although formally conceivable and intellectually appealing, I am not sure this view will prove to be a useful representation of the evolutionary process.

First, I note that FUEs are defined operationally: we essentially call FUE a maximal (in size) genetic element whose evolutionary history can be unambiguously represented by a single, bifurcating tree. Then, page 12, it is said that FUEs are "subject to selection independently of other FUEs". This is an important, questionable shift, which I think reveals a fundamental problem we have with HGT: the decoupling of transmission and selection (of the genetic material). Genomes (organisms), not genes, are the competing entities, between which natural selection operates, but genes can sometimes be transmitted between genomes.

Authors' response: *There are several fundamental issues here, and there might be some genuine conceptual disagreement. We strongly believe that selection is a multilevel phenomenon that does affect individual FUEs although it certainly also acts at ensembles of FUEs, up to the organismal and, possibly, higher levels. Furthermore, it seems important to mephasize that we do not define a FUE as «genetic element whose evolutionary history can be unambiguously represented by a single, bifurcating tree». What we argue is that tree-like evolution is an inalienable property of FUEs. Furthermore, we also believe that there is a strong, intrinsic link between transmission and selection of genetic elements that works both ways: elements that are subject to selection are most likely to be transmitted, and elements that have a high transmission potential are the most selectable ones*.

The logical but somewhat extreme viewpoint taken in this manuscript, in which transmission units are called evolutionary units (irrespective of selection units), appears to me not devoid of problems. For instance, it calls "evolving" items (genes) that most biologists would not call "living", although evolution is central in, e.g., Maynard-Smith's definition of life. The FUE concept also weakens the importance of species, and consequently taxinomy and systematics, and tends to separate evolution from ecology, and micro- from macro-evolution.

Authors' response: *As argued above, transmission units are not actually separated from evolutionary units under our concept. On the contrary, there is a strong connection between the two*. *With regard to definitions of life and what most biologists will or will not call "living", we cannot bring ourselves to care. We certainly do not claim that FUEs are "alive" (or otherwise, "not alive"). They are essential units of the biological world, and that is more than enough. A recent debate on whether viruses are "alive or not" and how relevant the answer to that question is to our understanding of evolution provides a perspective on this issue *[69-71].

Denying species the role of evolving units because of the existence of phylogenetic problems appears questionable to me. Species are typically defined irrespective of phylogeny. Nobody would deny Homo sapiens the species status, although we are phylogenetically the combination of two ancestral species (a proto-eukaryote and a proto-mitochondrion). The reason why we have problems defining species in prokaryotes is lack of sexual reproduction, not HGTs.

Authors' response: *On this issue there seems to be no fundamental disagreement but rather a semantic one. Indeed, species can be meaningfully defined only for life forms with regular sexual reproduction and the resulting reproductive isolation (from other species). Accordingly, prokaryotes do not "really" form species *[43]. *Animals certainly do form species (sensu Mayr). There is no reason to deny Homo sapiens the species status but it is useful to remember that this species goes back only some 5-6 million years not to the time of the mitochondrial symbiogenesis*.

8. Unnecessary, unimportant tree of life

I would like to know more about the prevalence of HGT before adopting this conclusion (and see ref 62 for a detailed version of Vincent Daubin's and my opinion on the subject). Let me just recall that the proportion of genes whose history has been affected by HGT is not a good measure of how much HGT blurs the species tree, since this proportion will mechanically increase as you add species (and one cannot reasonably argue he knows less and less about the species tree as he includes more and more data). Imagine a data set made of 1000 genes in 10000 species, in which every gene tree is distant only a single HGT from the consensus tree. I would be happy to draw this "tree of 0%", and call it the tree of life (and use it to map and study HGT events), even though not a single gene fully agrees with it. We need better measures of the phylogenetic impact of HGT.

Authors' response: *This seems to be a difference of degree not really kind. We agree that there is a central trend in the Forest of Life, and one may choose to denote it the tree of life although we are reluctant to do so. But we also know that the topologies of the trees in the FOL are highly inconsistent and that there is a substantial fraction of genes for which HGT is common *[66]. *Of course, we are not going to debate the point about the need to better measure the phylogenetic impact of HGT - yes, certainly, it needs to be measured better*.

### Reviewer 4: William Martin, University of Duesseldorf

I'm not terribly keen on this paper. It first sets up a tree of life crisis: is the history of life shaped like a tree in the sense that Darwin envisaged or is it not because unbeknownst to Darwin different genes have different histories. It then purports to solve the tree of life crisis by conjuring up a fundamental unit of evolution (FUE) that is defined as a thing having "a substantial degree of evolutionary independence" (a very soft definition that could apply to a nucleotide or a human being, depending upon the meaning of substantial) and taken here to mean nucleotide stretches of some length. They point out that FUEs, if energetically coerced to replicate and mutate in the absence of recombination, generate tree-like structures of genealogy over time, which I think we already knew. Then they stride into the conclusions that the history of life is properly represented by the sum of all FUE-trees, giving the forest of life (the FOL), but without providing such a graphical representation or approximation thereof, and surmise that "a species or organismal tree is not a fundamental concept". My beef is this: By declaring the object of debate (an organismal tree) not to be a fundamental concept, they do not contribute to the debate, but open up a different one that detracts from the one at hand. For example, I could say "no FUE can go about its replication business without some source of energy being tapped, such that replication is not a fundamental concept, energy is the issue," but that does not help us to review this paper, it just opens up a different issue. In my view, that is what the present paper does in the context of the present debate.

Moving swiftly to the final passage of the text, with the content of which I completely disagree, we see that it says: "Darwin was entirely correct in his belief that a tree is an accurate depiction of the evolutionary process but, for obvious reasons, he was unable to correctly identify the fundamental unit of evolution." I think I can discern what the first part of that sentence means in the context of this paper, but it is only true in the absence of recombination, and the question of what the fundamental units of evolution are is distinct from what people are debating, namely is there a tree of life or is there not? Moreover, no gene-like molecule (FUE) replicates by itself, it needs a cell to do that, so that the salient argument of what the units of evolution are fails, in my view.

One aspect of the current debate is nicely summed up in a passage on page 4025 of Galtier and Daubin [65], where they write:

*"Recently, several authors have questioned the usefulness of the species tree in prokaryote evolutionary genomics, based on the observation that only a tiny fraction (1% or less) of reconstructed gene trees are congruent with the reconstructed species tree (Dagan & Martin 2006; Bapteste et al. 2008). This, we think, is not a correct argument. In our view, the species tree could still be a useful concept even if incongruent with every gene tree, as we now discuss in more detail*.

Owing to incomplete lineage sorting, roughly 30 per cent of human genes do not support the (gorilla, (human, chimpanzee)) topology (Hobbolt et al. 2007). This problem must concern other recently diverged triplets of primate species. If we think of a dataset made of 10 such triplets (plus other species), then only 3 per cent (0.710) of true gene trees will be identical to the species tree. As we add more species, and more triplets affected by incomplete lineage sorting, this percentage decreases. Does this mean that the primate species tree is useless? Obviously not."

The foregoing quote discusses prokaryote genome evolution and lineage sorting in primate gene divergence in the same breath, as if they were the same process and subject to the same mechanisms. They are not. Meiosis is different from conjugation, transduction, natural competence, gene transfer agents as in *Rhodobacter*, and integrons and has different consequences over geological time. Some of us are projecting our views of evolution as obtained from the study of vertebrate phylogeny, which is tree-like, onto prokaryote evolution, which is not. In doing so, we are forcing the data from prokaryotes into the conceptual straightjacket of a tree, or filtering the observations from genomes so that only those that look tree-like come to be registered for interpretation. A better distinction needs to be made in this debate between eukaryotes, which undergo reciprocal recombination, and prokaryotes, which do not.

And the present paper? The present paper calls the FOL the solution and asserts that the search for the FOL is the grail, and that "the quest for an all-encompassing tree is futile". Well, in the paper where the FOL is presented [66], Koonin and Wolf surmise that there is some central trend and show trees of life. I see some inconsistency there, but no matter.

Authors' response: *In our opinion, no inconsistency whatever. We show trees that reflect the central trend that objectively exists in the FOL *[66]* and this is how we discuss the issue here. One may choose to call describe this central trend "a tree of life" but we at least find this more misleading than helpful given the difference from the traditional "species tree" definition*.

As I see it, the current tree of life debate, whether crisis or not, is about the things that Ford Doolittle has recently summed up elsewhere [72]. My own views on the issue are largely similar to Doolittle's and are to some extent expressed in a different paper having many authors that is also submitted to Biology Direct at this time [73].
